# The Research of Feature Extraction Method of Liver Pathological Image Based on Multispatial Mapping and Statistical Properties

**DOI:** 10.1155/2016/8420350

**Published:** 2016-02-28

**Authors:** Huiling Liu, Huiyan Jiang, Bingbing Xia, Dehui Yi

**Affiliations:** ^1^Software College, Northeastern University, Shenyang 110819, China; ^2^The Department of Hepatobiliary Surgery, The First Affiliated Hospital of China Medical University, Shenyang 110001, China

## Abstract

We propose a new feature extraction method of liver pathological image based on multispatial mapping and statistical properties. For liver pathological images of Hematein Eosin staining, the image of R and B channels can reflect the sensitivity of liver pathological images better, while the entropy space and Local Binary Pattern (LBP) space can reflect the texture features of the image better. To obtain the more comprehensive information, we map liver pathological images to the entropy space, LBP space, R space, and B space. The traditional Higher Order Local Autocorrelation Coefficients (HLAC) cannot reflect the overall information of the image, so we propose an average correction HLAC feature. We calculate the statistical properties and the average gray value of pathological images and then update the current pixel value as the absolute value of the difference between the current pixel gray value and the average gray value, which can be more sensitive to the gray value changes of pathological images. Lastly the HLAC template is used to calculate the features of the updated image. The experiment results show that the improved features of the multispatial mapping have the better classification performance for the liver cancer.

## 1. Introduction

Computer aided diagnosis (CAD) technology is to help designers design work by computer and its graphics equipment. Analyzing the pathological images automatically or semiautomatically by computer aided diagnosis technology can assist doctors to get a quick and accurate diagnosis result. Combing the CAD and the experience of expert can reduce the misdiagnosis result from the fatigue or subjective consciousness of doctors and can also reduce the labor cost. In recent years, many researchers have made a thorough study on medical image classification. Ramírez et al. [1] recognized the brain images of patients with Alzheimer's disease based on neural network. Wolz et al. [2] proposed a segmentation algorithm based on hierarchical position labeling and weighting algorithm for multi organs. They experimented on 100 CT images, and the overlap rate of liver, spleen, pancreas, and kidney was 94%, 91%, 66%, and 94%, respectively. Plissiti et al. [3] segmented the nucleus of the cervix slice images by shape, texture, and intensity features, which obtained a good segmentation result.

Liver cancer is the malignant tumor that threatens the life of human beings. The current methods for liver cancer diagnosis are CT examination, magnetic resonance imaging, ultrasonic imaging, and X-ray examination. And the ultrasound imaging is the best method for screening regularly and location of lesions [4–6]. These methods need artificial observation, and the early symptoms of liver cancer are not obvious, which increases the difficulties of the diagnosis for doctors. Therefore, applying the CAD to liver cancer diagnosis has the huge significance for recognizing liver cancer earlier.

In recent years, applying the CAD to liver cancer recognition has become a hot research direction. Lee and Hsieh [7] proposed a robust method to calculate the fractal dimension, which used fractal dimension and M band wavelet transform coefficients as features, realizing the recognition of ultrasonic liver images. Hao and Zhang [8] extracted the first-order statistical feature, gray level cooccurrence matrix feature, and gray stroke matrix feature of normal liver CT image and primary liver cancer CT images and then selected features by *t*-test method. Lastly, they recognized the liver CT images by BP neural network classifier according to the selected features. Wu et al. [9] discussed the feasibility of multiresolution feature selection and its application to the classification of ultrasonic liver images. Kondo et al. [10] proposed a new algorithm combining hybrid GMDH-type neural network and artificial intelligence. The innovation of this improved neural network algorithm is that it has a feedback loop, which can be used to determine the features of medical images, so as to reduce the misdiagnosis rate of liver cell carcinoma. Yang et al. [11] proposed a classification method of liver pathological images based on voting optimization. This method built classification model based on random forest and made the voting decision optimization by statistical analyses method, which optimized the classification model. The optimized classification model obtained the good classification performance.

We propose a new feature extraction method of liver pathological image based on multispatial mapping and statistical properties. ACHLAC is based on the traditional HLAC, for each pixel, making the update correction by the average gray value of the whole image before the template processing, which makes up the deficiency of traditional HLAC which cannot reflect the change of local gray value well. The process of classification of pathological images is as follows: firstly, we map the liver pathological images into entropy space, LBP space, R space, and B space and extract the ACHLAC features and gray level cooccurrence matrix (GLCM) features. Then we cascade the features extracted from different space and select features by kernel principal component analysis (KPCA). Lastly, we use the SVM to classify the liver pathological images according to the selected features. The experiment results show that the ACHLAC of multispatial mapping has better classification performance than the original HLAC and extended HLAC, which has the higher classification accuracy.

## 2. Method

### 2.1. Multispatial Mapping

Comparing the image features extracted from a single RGB space, mapping the image to multiple spaces can get more comprehensive information. HE staining refers to Hematein Eosin staining; Hematein stain is alkaline, which makes the chromatin in the nuclear and the ribosomal in the cytoplasm present purple blue. Eosin is acid dyes, which makes the components in the cytoplasm and extracellular present red. So, for the liver pathological images stained by HE, channel R and channel B contain more information, which can be more effective to classify the liver cancer. So we map the liver pathological images to R space and B space.

Because the entropy space and LBP space can reflect the texture information of images better, we also map the images to entropy space and LBP space. The calculation of mapping image to entropy space is as follows. The histogram of gray image to 0–255 is calculated firstly, and then the probability of gray value *i* can be calculated as *p*_*i*_. The gray entropy of image is calculated by

(1) $$\begin{array}{c} \text{entropy}=\overset{255}{\underset{i=0}{\sum }}p_{i}\log p_{i}. \end{array}$$

The calculation of mapping image to LBP space is as follows. Firstly, sample the neighbor pixels by 3 × 3 template and compare the gray value of each neighbor pixel to the central pixel. If the gray value of neighbor pixel is more than the central pixel's, we label the neighbor pixel as 1; otherwise we label the neighbor pixel as 0. Then we present the neighbor pixel as a binary sequence and turn it to a decimal number as the gray value of the central pixel. Update every pixel of image, and the updated image is called image of LBP space. The image of R space and B space is the image of obtaining R channel and B channel, respectively.

**Figure 1:**
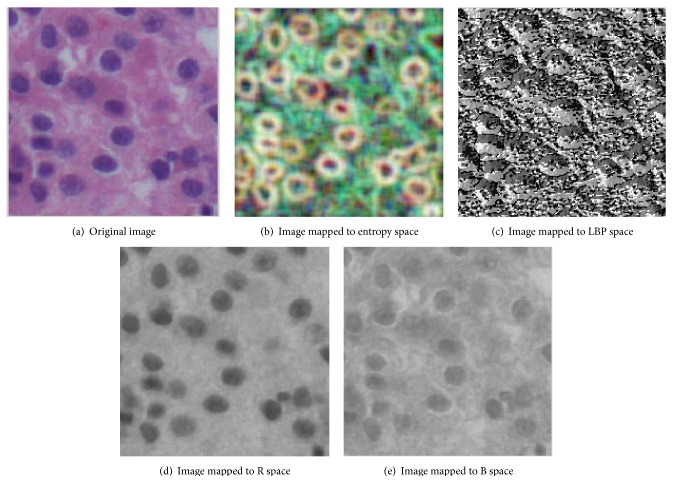
Images mapped to different spaces.

We map the liver pathological images to entropy space, LBP space, R space, and B space. Extract features from four spaces, respectively, and cascade the features, which can get the more comprehensive and representative feature.  Figure 1 shows the result images mapped to entropy space, LBP space, R space, and B space, respectively.

### 2.2. GLCM

Gray level cooccurrence matrix (GLCM) is used to describe texture feature, and it can reflect the characteristics of the image from the gray level cooccurrence matrixes.

In our experiment, features are extracted from four matrixes, and the four matrixes are from four directions *θ* = {0°, 45°, 90°, 135°}. For every matrix, four features are extracted: energy, contrast, correlation, and homogeneity.

Energy is the sum of squares of each element in the GLCM, which reflects the uniformity of the gray distribution of the image. Contrast reflects the texture depth extent of striation and the clarity of the image. Correlation represents the gray level similarity extent of rows or cols of GLCM, which reflects the consistency of the image texture. Homogeneity reflects the uniformity of the image.

### 2.3. HLAC

Higher Order Local Autocorrelation Coefficient (HLAC) [12] is proposed by professor Otsu from Tokyo University at IAPR in 1988. It shifts the higher order adaptive function and limits the changes of order, extracting higher-order statistical feature of binary image by template matching. HLAC features have shift invariance and have good performance in recognition field.

The local autocorrelation function of order *N* and pattern *P* is defined by (2) [13]. Consider

(2) xfNa1,a2,…,aN=∫Pfrfr+a1⋯fr+aNdr,

where *x* is the statistical feature of image, *r* is the position vector of current pixel, *f*(*r*) is the gray value of position *r*, and *a*_1_, *a*_2_,…, *a*_*N*_ is the offsets.

**Figure 2:**
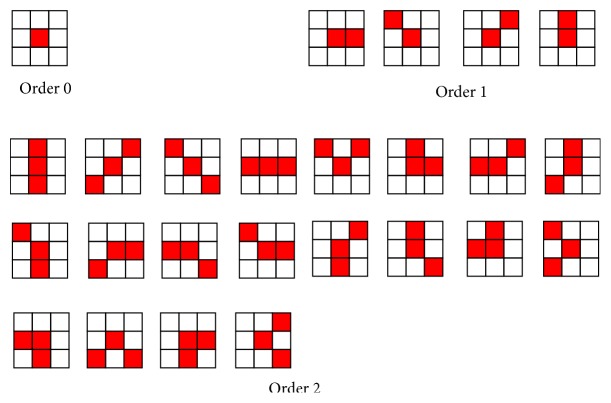
HLAC templates of order 2, with pattern 3 × 3 area.

With the increasing of the order and the offsets of the local autocorrelation function, the number of extracted features is increasing rapidly. For example, for the order is 8 and the offset is limited to area 3 × 3, it can get 223 local autocorrelation templates after removing the same pattern after shift transformation, corresponding to 223 features. For the order is 2 and the offset is limited to 5 × 5 area, it can get 205 local autocorrelation templates after removing the same pattern after shift transformation, corresponding to 205 features. Because the increase of order and offset will make the number of features increase rapidly, practically, the order is always limited to 2, and the offset is limited to 3 × 3 area. After shift transformation, it can get 25 local autocorrelation templates after removing the same pattern, which are shown in Figure 2. The red area is the labeled region of template, and the white area is the unlabeled region of template.

The method of calculating 25 feature vectors is as follows: traverse each pixel of pathological image and match it by the template of Figure 2. Multiply the gray value of labeled points in the template, and accumulate these products together, which is the final feature vector. The calculation formula of is shown as

(3) $$\begin{array}{c} x_{1}=\underset{i}{\sum }\underset{j}{\sum }f\left(\;i,j\;\right),\\ x_{2}=\underset{i}{\sum }\underset{j}{\sum }f\left(\;i,j\;\right)f\left(\;i+1,j\;\right),\\ x_{3}=\underset{i}{\sum }\underset{j}{\sum }f\left(\;i,j\;\right)f\left(\;i-1,j-1\;\right),\\ \vdots \\ x_{25}=\underset{i}{\sum }\underset{j}{\sum }f\left(\;i,j\;\right)f\left(\;i+1,j-1\;\right)f\left(\;i+1,j+1\;\right). \end{array}$$

Because of the limitation of the traditional HLAC function, we can improve its robustness by improving the HLAC template. Some research improved the HLAC templates by changing the order and window size [14], which improve the effectiveness of the feature.

**Figure 3:**
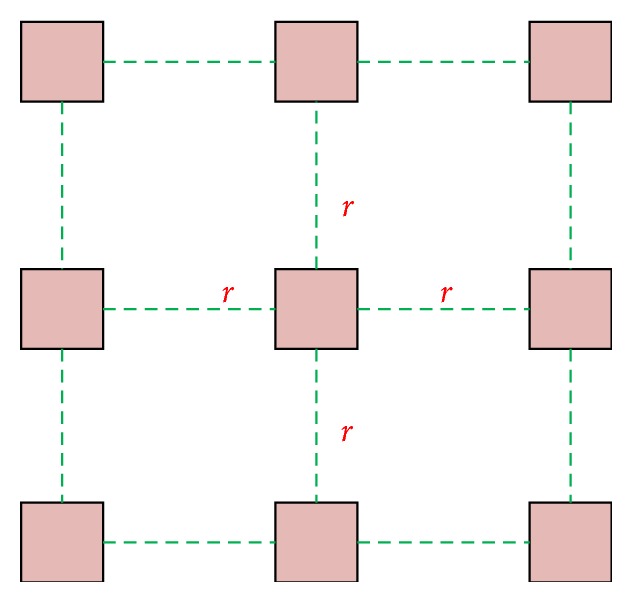
The extended process of HLAC template.

Extending the size of the window can increase the correlation of more data and then improve the robustness of algorithm. For the size of window is 5 × 5, the number of HLAC templates is as high as 205, which is much bigger than the number of HLAC templates of 3 × 3 window. If one creates HLAC templates in this way, it not only has more strict requirement for the feature selection but also increases the computational burden. Therefore, when extending the size of window, the size of offset is extended proportionally, which can extend the size of window without increasing the number of HLAC templates. This method, which can extend HLAC template to any radius, is extended HLAC method. The extended process of HLAC template is shown in Figure 3. The extended HLAC template size is *r* × *r*, because the size is extended proportionally, we can also label pixels as Figure 2; we can also say the extended HLAC is using the labeled templates of traditional HLAC, but the pixels of template is extended to radius *r*. We label pixels on nine pixels of Figure 3, and the label pattern is the same as Figure 2. In our experiment, the extended radius is 5, which can get the best classification accuracy.

### 2.4. Average Correction HLAC

Traditional HLAC multiply the gray value of labeled pixel in the template directly, which cannot reflect the information of image comprehensively. For example, the gray value of neighbor pixels in template are 2 and 125 and 15 and 16, and the multiplying result is 250 and 240, respectively. The multiplying result is similar, but the gray value distribution of the pixels is very different. The local variance of the former is significantly higher than that of the latter. The local variance of abnormal liver pathological image is higher, so the former has higher possibility as abnormal liver pathological image. However, we cannot recognize the difference between the former and latter according to the traditional HLAC. So multiplying directly may lose some information.

Aimed at the above disadvantages of traditional HLAC method, we propose an average correction HLAC method, ACHLAC method. Firstly, calculate the average gray value of the whole image. Then, for each pixel, calculate the absolute value of the difference between the current pixel gray value and the average gray value and update the current pixel. The updated image can reflect the variation of gray value better. Lastly, calculate the HLAC features according to updated image. The traditional HLAC considers local information only, and the improved ACHLAC is combining the local gray value and the whole average gray value, which can not only reflect local information but also reflect local changes of gray value. ACHLAC can make up the deficiency of HLAC in a certain extent.

To avoid the calculation differences result from the size of images, we make a normalized processing for extracted ACHLAC features. Consider the max of *x*_1_, *x*_2_,…, *x*_25_ as max, and divide each element and max, which can normalize the ACHLAC to [0, 1].

For the ACHLAC method, order is 2 and the offset is limited to 3 × 3 region. The pseudocode of ACHLAC feature extraction is shown in Algorithm 1, where abs() is the function of absolute value, pixel is the current pixel, *p*_*i*_ is the *i*th template in Figure 2, and max() is the function of maximum.

### 2.5. Classification of Liver Pathological Image

**Figure 4:**
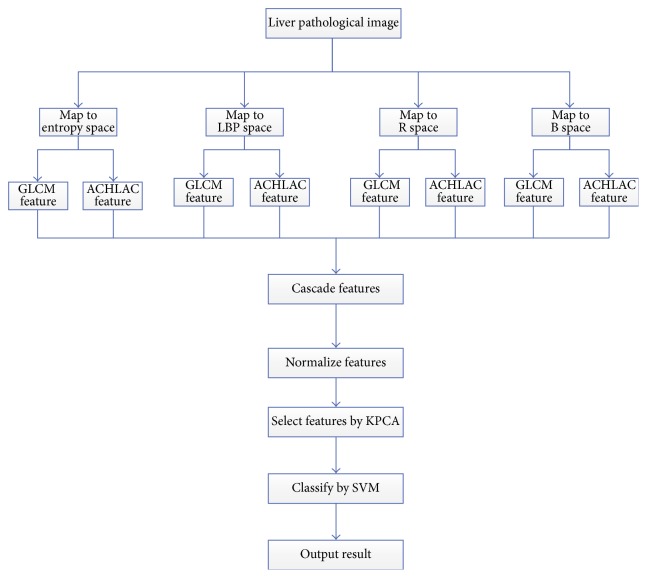
Classification of liver pathological image.

Our classification process is shown in Figure 4:

1. Input the liver pathological image.
2. Map the liver pathological image to entropy space, LBP space, R space, and B space.
3. Extract 25 ACHLAC features and 16 GLCM features in every space, which can get 41 features in every space, and then cascade the four space features, which is 164 features in total.
4. Select features by KPCA, and the dimension of features is decreased to 7 after KPCA.
5. Classify the images by SVM according to the selected features.
6. Output the result, normal liver pathological image or abnormal liver pathological image.

The training procedure is similar to classification procedure. For the training image, map it to entropy space, LBP space, R space, and B space and extract GLCM feature and ACHLAC feature. Then cascade the features of four spaces together, and select features by KPCA after normalizing features. After that, the features after selecting by KPCA are used to train the classification model by SVM. When the classification model is finished, we can use it to classify the testing images.

## 3. Experiment

### 3.1. Experiment Data

The liver pathological images in the experiment are provided by a hospital in Shenyang, China. The resolution of image is 160 × 120. Select 599 images as train data; the rest 560 images are used as test data. The train data contains 264 normal liver pathological images and 225 abnormal liver pathological images. The test data contains 256 normal liver pathological images and 304 abnormal liver pathological images. The experiment data is shown in Table 1.

### 3.2. Evaluation Criteria

There are much evaluation criteria of classifier performance, and the common evaluation criteria are accuracy, true positive rate, false positive rate, true negative rate, and false negative rate. The calculation of these evaluation criteria is based on the hybrid matrix, which is shown in Table 2.

In the hybrid matrix, the number of positive samples that is classified correctly is TP, the number of negative samples that is classified correctly is TN, the number of negative samples that is classified wrongly is FN, and the number of positive samples that is classified wrongly is FP. The number of practical positives is P, and the number of practical negatives is N. The total number of samples is *C* = P + N. The calculation formulas of evaluation criteria we used are shown as follows.

False positive rate, FPR: it is practical negative samples which are predicted as positive samples:

(4) $$\begin{array}{c} FPR=\frac{FP}{N}. \end{array}$$

True negative rate, TNR: it is practical negative samples which are predicted as negative samples:

(5) $$\begin{array}{c} TNR=\frac{TN}{N}. \end{array}$$

True positive rate, TPR: it is practical positive samples which are predicted as positive samples:

(6) $$\begin{array}{c} TPR=\frac{TP}{P}. \end{array}$$

False negative rate, FNR: it is practical positive samples which are predicted as negative samples:

(7) $$\begin{array}{c} FNR=\frac{FN}{P}. \end{array}$$

Accuracy: it is percentage of classifying correctly samples:

(8) $$\begin{array}{c} \text{Accuracy}=\frac{TP+TN}{C}. \end{array}$$

Because the FPR and TNR and FNR and TPR are complementary, this means the sum of FPR and TNR is 1, and the sum of FNR and TPR is 1. We select accuracy, TNR, and TPR as the evaluation criteria.

### 3.3. Result Analysis

**Figure 5:**
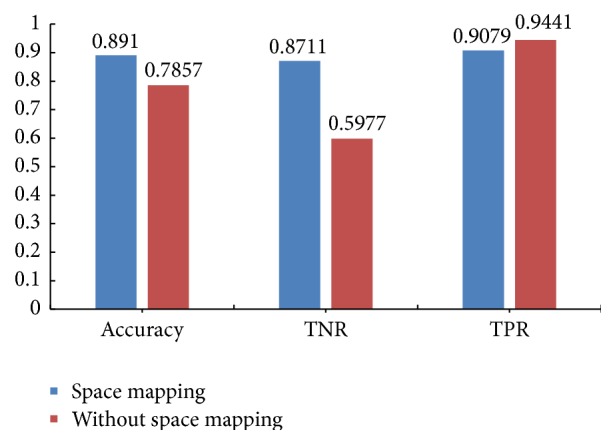
The experiment result of under space mapping and without space mapping.

Firstly, we experiment on the effective of space mapping. We calculate the classification accuracy, TNR, and TPR under space mapping and without space mapping, respectively. The experiment result is shown in Figure 5.

Figure 5 shows that the classification accuracy of under space mapping is higher than that of without space mapping. Although the TPR of without space mapping is better, the TNR is too low, which results in the poor classification performance. The TPR and TNR of under space mapping are good, so we can learn that mapping the liver pathological image to different space can improve the classification performance.

**Figure 6:**
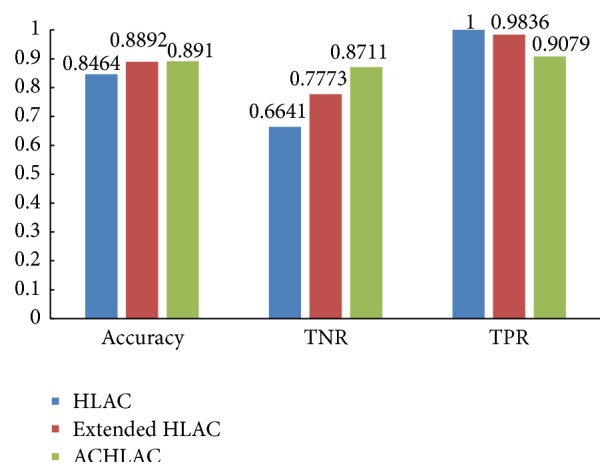
Contrast experiment using ACHLAC and GLCM features.

Then we validate the effectiveness of ACHLAC. In the experiment, we compare the classification performance between the ACHLAC and original HLAC. To verify the ACHLAC better, we also experiment on extended HLAC, which is an advanced and effective improved HLAC method. The experiment result is shown in Figure 6.

Figure 6 shows that the ACHLAC features obtain better classification performance compared to original HLAC and extended HLAC. Although the TPR of ACHLAC is a little lower than original HLAC and extended HLAC, the TNR is obviously higher than other methods. In a comprehensive view, the improved ACHLAC has better classification performance.

**Figure 7:**
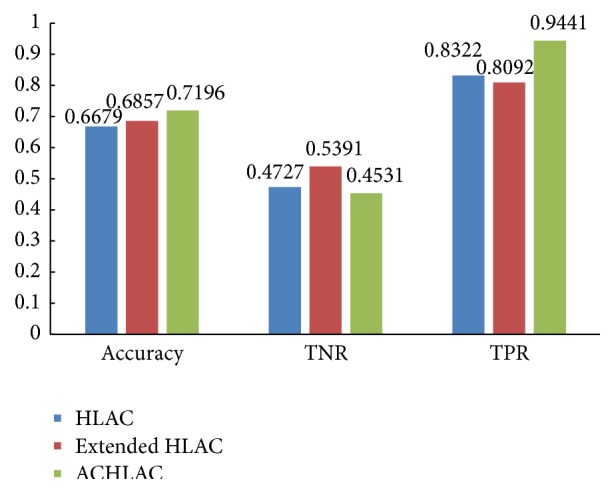
Contrast experiment using only ACHLAC features.

To avoid the influence of GLCM features, we verify the classification performance only by ACHLAC features. We also compare the experiment result of ACHLAC to that of the original HLAC and extended HLAC. In the experiment, we classify the images by SVM only according to ACHLAC, HLAC, and extended HLAC. The experiment result is shown in Figure 7.

Figure 7 shows that the liver pathological images can be classified only by ACHLAC features. ACHLAC has higher classification accuracy compared to original HLAC and extended HLAC, and ACHLAC is more sensitive to abnormal liver pathological images, which can recognize the abnormal liver pathological images better.

**Figure 8:**
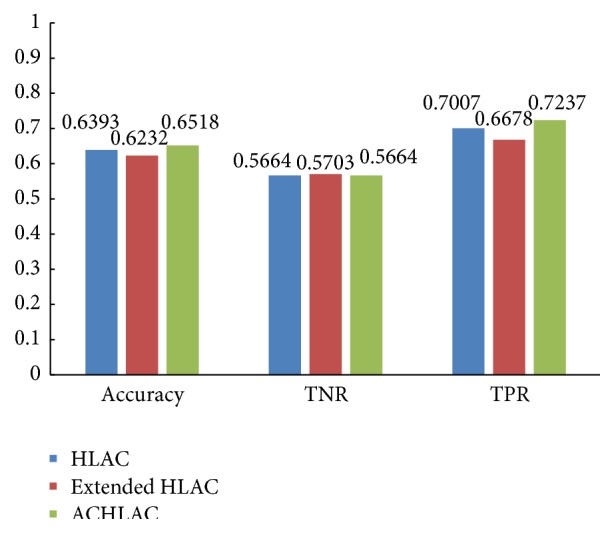
Contrast experiment using RF classifier.

**Figure 9:**
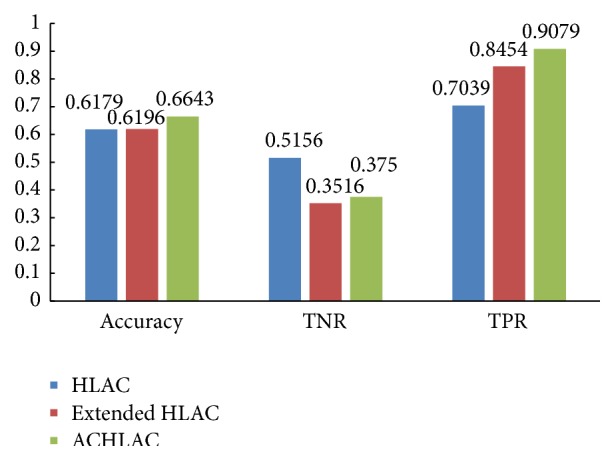
Contrast experiment using kNN classifier.

To prove that the ACHLAC features also have good classification performance by other classifiers, we also use the random forest (RF) classifier and kNN classifier to classify the liver pathological images. In the experiment, only features related to HLAC are used.  Figure 8 shows the experiment result using RF classifier, and Figure 9 shows the experiment result using kNN classifier.

From Figures 8 and 9, we can learn that the proposed ACHLAC has better classification accuracy than original HLAC and extended HLAC no matter using RF classifier or kNN classifier, which proves the universality of ACHLAC. ACHLAC is more sensitive to abnormal liver pathological images, which is more beneficial to recognize liver cancer.

From the contrast experiment, the effective and universality of ACHLAC are proved. It will get the higher classification accuracy combining with other features.

## 4. Conclusion

We propose a new feature extraction method of liver pathological image based on multispatial mapping and statistical properties. Other than extracting features in single RGB space, we map the liver pathological images to entropy space, LBP space, R space, and B space. Extract features under different space, and cascade them finally. Traditional HLAC, which multiplies the labeled pixel of template directly, cannot reflect the local changes of gray value well. We propose an average correction HLAC feature, which can reflect the local changes of gray value well, making up the disadvantage of traditional HLAC. From the contrast experiment, it is proved that the proposed ACHLAC with multispatial mapping has good classification performance.

## Figures and Tables

**Algorithm 1:**
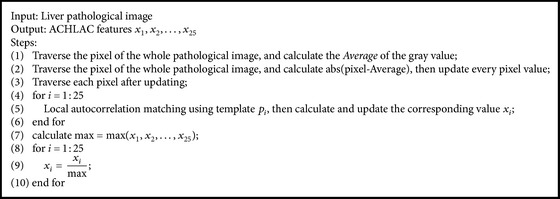
ACHLAC feature extraction.

**Table 1:** Experiment data.

	Normal (negative)	Abnormal (positive)	Total
Train data	264	335	599
Test data	256	304	560
Total	520	639	1159

**Table 2:** Hybrid matrix.

	Predict positive	Predict negative
Practical positive (P)	True positive (TP)	False negative (FN)
Practical negative (N)	False positive (FP)	True negative (TN)
